# Cancer Vaccines in Ovarian Cancer: How Can We Improve?

**DOI:** 10.3390/biomedicines4020010

**Published:** 2016-05-03

**Authors:** Silvia Martin Lluesma, Anita Wolfer, Alexandre Harari, Lana E. Kandalaft

**Affiliations:** 1 Center of Experimental Therapeutics, Ludwig Center for Cancer Res, Department of Oncology, University of Lausanne, Lausanne 1011, Switzerland; Silvia.Martin-Lluesma@chuv.ch (S.M.L.); Alexandre.Harari@chuv.ch (A.H.); 2 Department of Oncology, University of Lausanne, Lausanne 1011, Switzerland; Anita.Wolfer@chuv.ch; 3 Ovarian Cancer Research Center, University of Pennsylvania, Philadelphia, PA 19104, USA

**Keywords:** ovarian cancer, cancer vaccines, dendritic cell vaccination

## Abstract

Epithelial ovarian cancer (EOC) is one important cause of gynecologic cancer-related death. Currently, the mainstay of ovarian cancer treatment consists of cytoreductive surgery and platinum-based chemotherapy (introduced 30 years ago) but, as the disease is usually diagnosed at an advanced stage, its prognosis remains very poor. Clearly, there is a critical need for new treatment options, and immunotherapy is one attractive alternative. Prophylactic vaccines for prevention of infectious diseases have led to major achievements, yet therapeutic cancer vaccines have shown consistently low efficacy in the past. However, as they are associated with minimal side effects or invasive procedures, efforts directed to improve their efficacy are being deployed, with Dendritic Cell (DC) vaccination strategies standing as one of the more promising options. On the other hand, recent advances in our understanding of immunological mechanisms have led to the development of successful strategies for the treatment of different cancers, such as immune checkpoint blockade strategies. Combining these strategies with DC vaccination approaches and introducing novel combinatorial designs must also be considered and evaluated. In this review, we will analyze past vaccination methods used in ovarian cancer, and we will provide different suggestions aiming to improve their efficacy in future trials.

## 1. Rationale for Immunotherapy in Ovarian Cancer

Epithelial ovarian cancer (EOC) is one important cause of gynecologic cancer-related death, with an overall five-year survival rate of ~45% and an overall 10-year survival rate of 35% in the USA [1]. Globally, it is estimated that 282,741 new cases would be diagnosed with ovarian cancer in the world in 2020, with about 66% of the cases affecting women aged <65 years [2]. Currently, the mainstay of ovarian cancer treatment consists of cytoreductive surgery and platinum-based chemotherapy (introduced 30 years ago) but, as the disease is usually diagnosed at an advanced stage, its prognosis remains poor, with an overall five-year survival rate of ~45%, which drops to 28% for invasive EOC diagnosed at stage IV [1]. There is a clear unmet need for developing new treatment approaches and a potentially attractive approach for ovarian cancer treatment is immunotherapy [3].

It was recently demonstrated that EOC is an immunogenic tumor that can be recognized by the host immune system [4]. Indeed, tumor reactive T cells and antibodies can be detected in the blood, tumor and ascites of EOC patients with advanced disease [5,6]. The tumor reactive T cells collected from patients harboring advanced ovarian cancer are oligoclonal, recognize autologous tumor-associated-antigens (TAAs) and exhibit tumor-specific cytolytic activity *in vitro* [7]. In fact, the observed frequency of serological responses to these antigens is variable according to tumor type, stage or grade [8]. However, in most tumors the tumor-reactive lymphocyte populations show impaired antitumor function *in vivo*, due to several mechanisms. Ovarian tumors present multiple mechanisms of immune evasion, which consequently reduce the efficacy of immunotherapy. Thus, recruitment of Tregs in the tumor microenvironment in ovarian carcinoma confers immune privilege and is associated with poor prognosis and reduced survival [9,10]. Other mechanisms include high expression of PD-L1 and IDO production, which are independently associated with poor prognosis in EOC [11,12,13]. Furthermore, it has been demonstrated that both local and systemic dysfunction of plasmacytoid Dendritic Cells (pDCs) play a critical role in the progression of ovarian cancer via induction of immune tolerance [14].

Immunotherapies include active, passive or immunomodulatory strategies; although some overlap exists among them [15]. Active strategies (such as vaccines or adoptive-cell therapies with autologous T-cells) aim to increase the ability of the patients’ own immune system to mount an immune response against their own tumor. The first type of immunotherapy used in oncology was “Coley’s toxin” [16], an adjuvant which induces *in vivo* vaccination. Vaccination strategies indeed represent an attractive approach in ovarian cancer treatment, as they are associated with minimal side effects or invasive procedures.

Vaccination strategies for the prevention of infectious diseases led to major medical successes, such as global smallpox eradication [17]. Prophylactic vaccines against viruses with known oncogenic potential (such as HPV or HBV) have a demonstrated effect in preventing cancer development [18], although they do not confer any benefit on pre-existing infections or lesions [19]. For this reason, therapeutic vaccines against established lesions have been developed, yet they have a long history of low efficacy that has created a negative image on immunotherapy. First, it should be kept in mind that prophylactic and therapeutic vaccines have different goals: in prophylactic vaccines, the aim is to trigger a good humoral response, so that effective antibodies are produced, able to bind to and inactivate the targeted pathogen when it enters the blood or mucosal surfaces. Yet, a therapeutic vaccine should be able to induce cell mediated immunity, so that immune cells are activated to identify and destroy their cellular targets in the affected tissues. Then, understanding the reasons for the limited efficacy of therapeutic vaccines to date will allow the development of new approaches that may aid to overcome the barriers to adequate anti-tumor activation. Some of these new strategies are currently being developed and tested in ovarian cancer with encouraging results.

## 2. Therapeutic Vaccines in Ovarian Cancer

Cancer vaccines can be classified in different categories, according to the method of choice to deliver the selected TAAs; thus, cell-based vaccines, peptide/protein, epigenetic, and genetic vaccines have been developed so far [20] (see Table 1). Vaccines can be given alone or in combination with different adjuvants, such as cytokines or other stimulatory factors [21]. Furthermore, different routes for immunization can be used, which contribute differently to immune cells activation: vaccine injections can be subcutaneous, intradermal, intranodal, intraperitoneal, or intravenous [22]. Finally, another consideration is the vaccination schedule, in terms of dose, number of injections and time, to initiate and maintain the appropriate immune response. More studies are warranted to determine which of those elements (alone and in combination) would offer optimal clinical efficacy [23]. Here, we will summarize the results obtained to date with all types of vaccination strategies in ovarian cancer with the intent to provide a general overview allowing undertaking more rational decisions (Table 1).

### 2.1. Cell-Based Vaccines

Cell-based vaccines can use DCs, which play a critical role in the interface between innate and adaptive immunity [59]. Their function consists in the uptake, process and presentation of antigenic peptides (either from pathogens or host-derived) to naïve T cells in peripheral tissues, and they constitute the most important antigen-presenting cell (APC) population for activating antitumor T-cell responses. For this reason, they are the most frequently used cellular therapeutics in clinical trials, also because DC vaccination has demonstrated a good safety profile, rarely presenting immune-related toxicities [60], and it is associated with preserved quality of life of cancer patients [61]. DCs can be given alone after cytotoxic therapy (either chemo or radiotherapy, which increase antigen availability *in vivo* [62]), or alternatively they can be loaded *ex vivo* with different antigens, such as whole tumor lysate, peptides, proteins, or genetic material delivering the desired antigen (transfected/electroporated DNA, RNA or transduced virus), prior to reinfusion into the patient.

The advantage of whole cells as a source for antigens is that they will present to the immune system the complete repertoire of TAAs from that particular tumor, including the specific neo-antigens, therefore predicting a better immune response [22]. In this direction, in a meta-analysis including about 1800 patients, those who were immunized with whole tumor vaccines had a significantly higher objective response (8.1%) than patients who were immunized with defined tumor antigens (3.6%) [63]. Ovarian cancer lends itself to surgical intervention during the course of disease, including for the recurrent setting. This enables whole tumor vaccination approaches, since tumor cells can be easily recovered through cytoreductive surgery. Indeed, we as well as others have shown clinical benefit in recurrent advanced ovarian cancer patients vaccinated with DCs loaded with whole tumor lysate [24,25]. In our study, we treated five recurrent ovarian cancer patients with DCs loaded with hypochlorus acid-oxidized whole tumor lysate (inducing primary necrosis and enhancing the immunogenicity of lysed tumor cells), administered intranodally. We observed potent T-cell responses against known ovarian tumor antigens, and two patients presented durable PFS of 24 months or more, with only few grade 1 toxicities. We believe this is an approach that warrants further exploration [25]; thereby, our group has continued this pilot clinical study in heavily pretreated recurrent ovarian cancer patients to test vaccine injected intranodally, alone or in combination with immunomodulatory therapy (NCT01132014).

An example of a whole tumor cell vaccine in ovarian cancer is gemogenovatucel-T (FANG vaccine); it consists of autologous tumor cells eletroporated with FANG vector, a plasmid encoding GM-CSF (granulocyte macrophage-colony stimulating factor; a potent stimulator of dendritic cell maturation) and a bi-shRNA targeting furin convertase, thereby downregulating endogenous immunosuppressive growth factors TGF-β1 and β2 [33]. This vaccine has been tested in a Phase I study including 27 patients with advanced or metastatic non-curable solid tumors of different types (5 ovarian cancer), and in 26 of 27 patients evaluable for tumor response, 23 achieved SD at Month 2 or later as best response. This vaccine is currently being tested in a Phase II/III study in women with stages III/IV high grade serous/endometrioid ovarian, fallopian tube or primary peritoneal cancer (NCT02346747).

Other approaches use DCs loaded with specific antigens, either peptides or proteins. The first validation of active immunotherapy as a viable approach to cancer treatment was the FDA approval of Provenge^®^ (sipuleucel-T) for advanced prostate cancer. Provenge^®^ is an autologous DC-based vaccine (developed by Dendreon), in which autologous peripheral blood mononuclear cells (PBMC) are matured with a cytokine and a tumor-derived differentiation agent, and then pulsed with a fusion protein composed of prostatic acid phosphatase (PAP; a tumor-associated differentiation antigen) linked to GM-CSF prior to reinfusion into patients [64]. In the pivotal Phase III trial of this vaccine in men with metastatic castration-resistant prostate cancer, clinical results showed little evidence of tumor shrinkage or delay in disease progression [65]. Nevertheless, a 4.1 month improvement in median survival was achieved (25.8 *vs.* 21.7 months), which was considered significant by the FDA in a patient population that has almost no other effective therapeutic option. A similar vaccine was prepared and tested by Dendreon (lapuleucel-T or Neuvenge), consisting of DCs loaded with a fusion protein of HER-2/neu linked to GM-CSF. This vaccine was targeted to patients with advanced adenocarcinomas of the breast, ovary, endometrium, or gastrointestinal tract with HER-2/neu positive tumors, yet in a Phase I study including 18 patients, only two (11%) experienced stable disease lasting more than 48 weeks [29].

### 2.2. Peptide/Protein-Based Vaccines

The broad use of autologous cancer vaccines, including DCs or whole tumor cells, is limited by both the availability of patient’s samples or specimens and the complex procedure of preparing individualized vaccines and, from this point of view, recombinant vaccines have a clear advantage. Peptide/protein-based vaccines are usually based on defined TAAs and administered together with an adjuvant or immune modulator to improve their uptake by endogenous DCs. Unfortunately, many initial attempts were compromised by a poor understanding of the mechanism of immunization: frequently patients were treated with vaccines consisting of short peptides, binding exactly to HLA class I molecules (usually HLA-A*02), which do not induce CD4+ T cells, resulting in short-lived CD8+ T cell responses, often even without an effective adjuvant [66]. A better understanding of the importance and function of DCs in stimulating T cell responses has led to a more rational design of current vaccines. For instance, the use of peptides (~20 mer) somewhat longer than the optimal MHC class I-binding molecules (10–12 mer), in the presence of a suitable DC-activating adjuvant, are thought to be more efficient at generating effector T cells, due to additional processing required for long peptides to allow loading in DC HLA molecules, leading to potential dual stimulation of CD4+ T as well as CD8+ T cells [67]. However, results observed with this type of long peptides are still far from clinically relevant [45,48,49,50]. On the other hand, the use of full length proteins (in principle available for complete processing by DCs) has given mixed results to date [42,46]

In ovarian cancer, many different peptides targeting HER-2/neu have been tested. HER-2/neu is a member of the epidermal growth factor receptor (HER/EGFR/ERBB) family, whose amplification in breast cancer is associated with increased aggressiveness, therefore becoming an important target of therapy for about 20%–30% of patients [68]. HER-2/neu overexpression/amplification has been reported in ovarian cancer [69], and consequently it was considered a potential target for cancer vaccination. However, in most studies using single or mixed HER-2/neu peptides, no immunogenicity was observed [38,39,40,41], and no clinical data was obtained. Other vaccine targets in ovarian cancers using peptides have been tested [43,44,45,47,48,49,50], generally with low efficacy in the clinic. The best results in ovarian cancer using peptide-based vaccines have been obtained using a personalized peptide vaccine (PPV), in which a mixture of 4 peptides (from a panel of 31) previously tested for immunity in each patient was admixed in Montanide ISA51VG and subcutaneously administrated. In this study [51], median survival time (MST) was 39.2 months in platinum-sensitive patients, compared to 16.2 months in platinum-resistant patients, whereas the corresponding values in standard of care patients are 18–30 months (platinum-sensitive) *vs.* 8–12 (platinum-resistant). Interestingly, it was observed that PPV induced not only peptide-specific immunological boosting in response to the vaccinated peptides but also promoted the spreading of immune responses to the other TAA-derived peptides, which together resulted in the prolongation of OS. Results here indicate that vaccination strategies in which the vaccine antigens are selected and administered based on the pre-existing host immunity before vaccination can prolong OS in advanced ovarian cancer patients.

### 2.3. Genetic Vaccines

Genetic vaccines (based on DNA, RNA or virus) can be used to induce expression *in vivo* of the selected TAAs in somatic (keratinocytes, myocytes) or dendritic cells infiltrating muscle or skin at the vaccination site, resulting in cross-priming or direct antigen-presentation to infiltrating T-cells. Genetic vaccines present the advantage of easy delivery of multiple antigens in one immunization and activation of various arms of immunity, in combination with cheaper and more standardized manufacturing [70].

In ovarian cancer, two viral vaccines have been tested so far. One group has focused on the “cancer-testis” antigen NY-ESO-1, engineered into vaccinia (rV) as prime and fowlpox (rF) as booster vaccination. In a Phase II study including 22 patients with advanced NY-ESO-1-expressing ovarian cancer at high risk of recurrence, showed encouraging results, as the median time to disease progression or recurrence was 21 months (95% CI, 16–29 months) and median OS was 48 months (CI non-estimable) [55]. A second genetic vaccine tested in ovarian cancer (PANVAC-C + PANVAC-V) is a Poxviral vaccine, in which the CEA-MUC1-TRICOM (B7.1, ICAM-1, LFA-3) was engineered into vaccinia (PANVAC-V) as prime and fowlpox (PANVAC-C) as booster vaccination. However, clinical results from a Phase I clinical trial including 25 patients with CEA- or MUC1-expressing metastatic cancers who had progressive disease following standard chemotherapy (three of them with ovarian cancer) showed limited evidence of clinical activity [53]. Currently, several clinical trials are testing different genetic vaccines in ovarian cancer treatments (see Table 2).

### 2.4. Epigenetic Vaccines

Glycosylation is the most diverse post-translational protein modification, playing a key role in a wide range of biological processes. It has been shown that epigenetic regulation of glycosyltransferases in cancer cells results in the creation of novel glycan structures [71,72,73], and this correlates with the fact that most tumor cells present altered glycosylation relative to the normal tissue from which they derive [74]. Altered glycosylation has been proposed to be one of the mechanisms used by cancer cells to evade the host immune response, since cellular presentation of glycopeptide and glycolipid antigens can be potent modulators of T cells [75]. Antiglycan vaccination strategies against tumors were proposed early [76], and they have subsequently been developed, mostly against the epithelial MUC1. However, due to poor immunogenicity, glycan-based vaccines need to be conjugated to helper T epitopes, such as keyhole limpet hemocyanin (KLH) [77].

In ovarian cancer, two antiglycan vaccines have been tested: one against Lewis(y) (Le^y^), and the Theratope vaccine, targeting Syalyl-Tn (STn). Le^y^ is a carbohydrate antigen overexpressed in ovarian cancer [78]. In a phase I study, 25 patients with persistent or recurrent ovarian, fallopian tube, or peritoneal cancer of any stage or grade at diagnosis were vaccinated with a synthetic Le^y^ pentasaccharide coupled to KLH carrier protein, together with the QS-21 immunological adjuvant. At a median of 18 months follow-up, 19 of 24 patients had either biochemical or measurable disease recurrence. The median TTP was six months (range 2–17 months), with five patients in CR at 18 months of follow-up. Theratope^®^ is a Sialyl-Tn—keyhole limpet hemocyanin (STn-KLH) vaccine that incorporates a synthetic STn antigen mimicking the unique tumor-associated STn carbohydrate, designed to stimulate tumor antigen-specific immune responses in patients with mucin-expressing tumors. STn expression is associated with a poor prognosis in metastatic breast [79] and ovarian cancer patients [80], among others. Theratope^®^ vaccine was tested in a Phase II/III trial including 70 patients with either advanced breast or ovarian cancer expressing mucin. Vaccination was performed after autologous stem cell transplantation, considering that patients with low tumor burden would be more likely to respond immunologically to a cancer vaccine. Interestingly, it was observed that vaccinated patients had a decrease in the risk for relapse and death (*p* = 0.07 and *p* = 0.10, respectively), as compared to patients who underwent transplantation during the same period, but were not vaccinated [56]. However, later on this vaccine failed to show any improvement in TTP nor patient survival in a big Phase III trial reported in 2011 for more than 1000 breast cancer patients, despite a vigorous and specific humoral response to the STn antigen [81]. A different epigenetic vaccine using the Globo H hexasaccharide 1 (Globo H) epitope linked to KLH is currently being tested in a Phase II study including patients with non-progressive epithelial ovarian, fallopian tube, or primary peritoneal cancer after cytoreductive surgery and platinum-based chemotherapy (NCT02132988). Globo H is an antigen that was identified on a variety of epithelial cell tumors of ovarian, gastric, pancreatic, endometrial, prostate, and lung (small cell and non-small cell) origin, and selected as potential target for cancer immunotherapy [82].

## 3. Improving Vaccination Strategies in Ovarian Cancer

Cancer vaccines have shown, in general, low therapeutic efficacy, probably associated with their inability to elicit a rapid and strong T-cell response, and this has generated a great deal of criticism [83]. Similar criticisms have been addressed specifically to DC-vaccination [84]. However, a systematic review analyzing all published clinical trials performed to document the proportion of patients who had an objective response rate after DC vaccination in melanoma, prostate cancer, malignant glioma, and renal cell carcinoma [85] demonstrated that, in melanoma, DC therapy had similar objective response (8.5%) than dacarbazine (standard of care), or ipilimumab (5%–15%). In prostate cancer patients, objective response rate was 7.1% after DC vaccination, similar to 10% of the population treated with conventional chemotherapeutic drugs. Comparably, objective response after DC therapy was 15.6% in patients with malignant glioma, and 11.5% in advanced RCC. Interestingly, in most studies using DC therapies an increase of at least 20% in overall survival has been documented [85], although many of these studies were early phase, in which survival was not the main endpoint. Therefore, given the positive toxicity profile associated with vaccines, it is important to identify novel strategies to increase their efficacy, ideally without inducing significant changes in toxicity. Basically, an ideal vaccination strategy should include: (i) an adequate mixture of immunogenic antigens (*ex vivo* or *in vivo*); (ii) either a selected maturation signal for the target DC population *in vivo*, or the targeted matured DC population loaded with those antigens *ex vivo*; (iii) a defined route of administration to enhance presentation to T cells; and (iv) at least one immunomodulatory agent aiming to reduce the immunosuppressor environment imposed by the tumor [66].

### 3.1. Choosing the Right Antigen

In ovarian cancer, the scarcity of well characterized tumor antigens and the elevated molecular heterogeneity of the disease [86] have represented an important limitation to finding an adequate target antigen for vaccination. Additionally, even when a defined target is known, and vaccination induces an immune response, evolution of the tumor selecting antigen-loss variants following vaccination by the process of immunoediting may hinder long-term benefit [43]. This proves that single-target immunization can result in tumor variants facilitating immune escape following initial response, and therefore vaccination with multiple defined antigens seems crucial for achieving significant clinical benefit.

Another possible reason for reduced efficacy is that, to date, most vaccines were targeted against defined non-mutated self-antigens [87]. Tumors express non-mutated self-antigens (the so-called “public” antigens) as a result of tissue or lineage-specific gene expression or gene deregulation induced by transformation. These tumor self-antigens are male germline antigens (such as NY-ESO-1), overexpressed antigens (as HER-2/neu) or tissue/lineage-specific antigens, which are shared both by the tumor and the tissue they originated from (as gp100 in melanoma). For most self-antigens, T cell reactivity against them is low by definition, due to the development of tolerance toward them, designed to avoid undesired autoimmune events.

On the other hand, tumors express a second class of TAAs that can be recognized by T cells: mutated neo-antigens. These neo-antigens result from the large number of mutations that happen in tumor cells as a consequence of their inherent genetic instability; therefore, they are fully tumor specific. Deep sequencing analysis of tumor cells has revealed that they harbor usually between 10 and few thousand private somatic mutations; most of these mutations are different even among tumors of the same histotype [88,89]. In contrast to self-antigens, T-cell reactivity towards neo-antigens shows a functional avidity similar to the avidity observed in anti-viral T-cells [90]. Furthermore, T-cell response against neo-antigens is not expected to induce any autoimmune toxicity against healthy tissues, making vaccination toward neo-antigens a very attractive option. However, neo-antigens are mostly patient-specific, since the individual mutations found in any part of the tumors are essentially distinct [91]. Currently, it is possible to identify the repertoire of mutation-derived epitopes (the mutanome) present in one tumor using state-of-the-art technology, such as next-generation sequencing (NGS) to produce a list of individual cancer mutations or prediction of epitopes binding to the patient’s haplotype using bioinformatics’ tools (e.g., NetMHC). This implies that, based on current knowledge, no vaccine can be designed to target shared neo-antigens in a large group of patients. Furthermore, identification of these “private-mutations” is currently laborious, and major automation is required for adaptation in routine practice [86]. In spite of this, a recent pilot clinical trial has demonstrated that vaccination with DCs pulsed with neo-antigen peptides is safe and effective in boosting neo-antigen-specific T-cells in 3 melanoma patients [92]. For these reasons, targeting neo-antigens is a potential source for vaccine improvement, and therefore several Phase I clinical trials have been started to test the concept of neo-antigen vaccines in cancer patients, in melanoma (NCT01970358, NCT02035956), glioblastoma (NCT02287428, NCT02510950, NCT02149225), and breast cancer (NCT02316457), using peptides (admixed with adjuvants) in some trials, or RNA to deliver the neo-antigens. This principle is also applicable to ovarian cancer.

Alternatively to the use of selected antigens, tumor antigens can be obtained directly from tumor cells or lysates, which will include both the public and the private antigens (the neo-antigens), without the hurdles of neo-antigen identification and preparation. In most cases, autologous tumor lysates used in vaccine preparation are subjected to multiple freeze–thaw cycles to induce primary necrosis of cancer cells. However, freeze–thaw induced necrosis has not demonstrated high immunogenicity, and it has been shown to even inhibit TLR-induced maturation and function of DCs [93]. It was also shown that induction of tumor cell stress before lysis could partially reverse lysate-induced DC suppression, and only DCs loaded with stressed lysates afforded protection against tumor challenge *in vivo*. Other approaches to improve the immunogenicity of whole tumor lysate vaccination have demonstrated some successes in the clinic. DC vaccines using whole lysate from irradiated tumor cells have been successfully implemented in clinical trials including melanoma, high-grade glioma, and prostate cancer patients [94,95,96]. Interestingly, in a clinical trial including 18 patients with relapsed B-cell lymphoma patients treated with a DC vaccine loaded with whole autologous tumor lysate treated by heat shock, γ-radiation, and UV ray, 6 patients (33%) showed clinical and immunological responses, which were positively correlated with the extent of calreticulin and heat shock protein 90 (HSP90) surface expression in the DC antigenic cargo [97]. Consistently, we are currently running two trials in ovarian cancer where we use autologous hypochlorous acid (HOCl)-oxidized whole tumor cells lysate vaccines after demonstrating in mouse models that HOCl-based oxidation induces primary necrosis of tumor cells, showing superior immunogenicity as compared to UVB irradiation and freeze–thaw cycles [25].

A potential alternative to increase antigen immunogenicity would be the combined use of DC vaccination with oncolytic viruses, given their potential to induce Immunogenic Cell Death (ICD). The beneficial effect of intratumoral delivery of oncolytic viruses prior to DC vaccination has been demonstrated in different murine tumor models [98,99]. These oncolytic viruses, active in ovarian cancer models, could be either directly injected into tumors prior to DC vaccination, or alternatively, oncolysates could be prepared from whole tumor cells, that could be loaded into DCs for more efficient vaccination. Finally, when using whole tumor cell extracts (either loading DCs or by direct vaccination), immunogenicity could be enhanced by adding specific molecules, such as a fusion protein of single-chain antibody variable fragment (scFv) mesothelin (MSLN), to Mycobacterium tuberculosis (MTB) heat shock protein 70 (HSP70), which is a potent immune activator able to stimulate monocytes and DCs, enhancing DC maturation and aggregation, and improving cross-priming of T cells. Intraperitoneal injection of this bifunctional fusion protein in murine models of ovarian cancer and mesothelioma increased tumor-specific CD8+ T-cell dependent tumor responses, significantly enhancing survival and slowing tumor growth [100].

### 3.2. Providing DC Maturation Signals to Enhance T Cell Activation

One important consideration regarding T cell activation is that, in the absence of adequate DC maturation signals, presentation of antigens to T cells may induce tolerance by production of regulatory T cells (Treg) [101,102,103]. Consequently, in vaccination strategies using peptides or proteins (single or mixtures; synthetic or whole cell lysates), either directly or pulsed onto DCs, an appropriate adjuvant must be incorporated, in order to provide the required activation/maturation signal to DCs that will allow them to differentiate, as well as to process and present tumor-antigen derived peptides to T cells [104,105]. For this reason, a number of different adjuvants are currently available, such as Toll-like receptor ligands, which play a key role in DC maturation [21]. However, the best adjuvant choice is still not defined. To this end, we are currently running a trial in ovarian cancer (NCT02452775) comparing OC-L vaccine alone with the addition of either Montanide (a water-in-oil emulsion possessing an immune stimulatory effect) or poly-ICLC alone (a TLR-3 agonist able to upregulate genes involved in innate immune pathways including IFN-α, IFN-β, IFN-γ upon administration in healthy volunteers [106]). In a previous pilot clinical study with ovarian cancer patients, we have also demonstrated that LPS-activated DCs produced high levels of Th-1 polarizing cytokines including IL-12p70 and CXCL10, as well as stimulated potent polyclonal tumor T cells in patients [25]. Other TLR agonists with potential interest in ovarian cancer vaccines are imiquimod, motolimod, or CpG-oligodeoxynucleotides (ODNs). Imiquimod is a TLR7 agonist approved by the FDA for topical use in basal cell skin cancer, which has been shown to induce IFN-α and other cytokines [107], as well as to efficiently activate DC maturation [108]; it has been successfully used as an adjuvant for NY-ESO-1 protein for treating metastatic melanoma [109]. Motolimod (VTX-2337) is a TLR8 agonist which is able to stimulate production of TNF-α and IL-12 from monocytes and myeloid DCs, and stimulates IFN-γ production from NK cells [110]; motolimod has been tested for dose finding in a clinical trial including patients with advanced solid tumors and lymphoma [111], and is currently being tested in patients with recurrent ovarian cancer, in combination with doxorubicin (NCT01666444).

Unmethylated cytosine-phosphate-guanine (CpG) dinucleotides, which are relatively common in bacterial and viral DNA but are suppressed and methylated in vertebrate DNA, are recognized by and able to activate TLR9. Several synthetic CpG-ODNs have been developed for cancer treatment [112]. In an ovarian preclinical model, CpG-ODNs have been tested as adjuvants in combination with DCs electroporated with whole tumor cell RNA, and found to enhance their efficacy [113]. Similarly, in a murine glioblastoma model, 55% of mice vaccinated with CpG/lysate combination demonstrated over two times greater median survival compared to mice treated with CpG only, tumor lysate only or no treatment (*p* < 0.05) [114]. Furthermore, CpG-ODNs can synergize with other TLR agonists to activate more than one DC subset, such as flagelin (a TLR5 agonist, leading to activation of pDCs and dermal DCs) [115], or poly-ICLC, a combination leading to Langerhans cells activation with strong production of IL-6 and IL-12 and enhanced antitumor immunity [116].

### 3.3. Targeting the Right DC

In DC vaccination strategies, another important consideration to increase immunogenicity is the choice of the DC subset. In most immunotherapy trials, monocyte-derived DCs are used, which are differentiated *ex vivo* with recombinant GM-CSF and IL-4 [117]. These mature DCs are efficient phagocytes of antigens, able to produce high IL-12 upon activation. Their major advantage is that they can be easily manipulated before infusing them into patients. However, the *ex vivo* production of these DCs is labor intensive and costly. One attractive alternative is to specifically target DCs *in vivo* with appropriate tumor antigens, activating them to elicit potent anti-tumor T cell responses. In the immune system, hematopoietic stem cells (HSCs) differentiate into common lymphoid progenitors (CLPs) and common myeloid progenitors (CMPs). CMPs subsequently differentiate into monocytes and pre-DCs in the bone marrow. Both monocytes and pre-DCs enter the blood and migrate to lymphoid organs and peripheral tissues, where they can differentiate into lymphoid DCs and tissue-resident DCs [118]. Differentiated DCs can be classified in different subsets, according to their phenotype, receptor expression, chemokine and cytokine production, tissue location, and the type of immune response they induce. Ideally, we could use specific adjuvants, as previously discussed, to selectively activate *in vivo* one or more DC subsets by their surface receptors. For instance, TLR7 and TLR9 are uniquely expressed in plasmacytoid DCs (pDCs) [119], and they can be activated to become potent secretors of IFN-α and -β with imiquimod and CpG-ODNs, respectively [120,121]. Langerhans cells present TLR3, and therefore can be activated using poly-ICLC; and dermal DCs present TLR4, thereby they can be activated with monophosphoril A (MPLA), which is a derivative of LPS with lower toxicity. Lastly, blood and lymph node DCs expressing TLR8 can be activated using motolimod. However, targeting DCs *in vivo* one would have less control over the quality and magnitude of the anti-tumor response. 

Additionally, selection of the administration route for vaccination can also have an effect in the effectiveness of DC maturation process. Traditionally, the routes used for vaccination against many infectious diseases have been subcutaneous (s.c.) and intramuscular (i.m.). However, different studies demonstrate that the intradermal (i.d.) route is more effective in inducing protective immunity, even inducing seroconversion in subjects unresponsive to i.m. HBV vaccination [122]. This is probably due to the fact that the dermis contains a much larger population of DCs compared to subcutaneous fat and muscles, as well as the extensive network of lymphatic vessels present in the dermis, which will favor an effective loading of antigen on DCs and their subsequent transport to draining lymph nodes for presentation to T cells. Alternatively, direct administration of antigens into the regional lymph node (intranodally, i.n.) is perhaps the most effective way to ensure maximum display of antigens to DCs, which will not have to migrate after antigen loading. Different routes of antigen-coding mRNA delivery has been analyzed in a mouse model, showing that following intranodal injection, resident DCs in the nodes selectively took up the mRNA, inducing potent CD4+ and CD8+ T cell responses after repeated i.n. injections in tumor-bearing mice, which was not observed with subcutaneous, intradermal, or near nodal administrations [123]. However, a different possibility is direct antigen administration to tumor-infiltrating DCs (TIDCs), which have been documented in different types of cancers, including ovarian cancer [124], although their function is not yet well characterized. Interestingly, in a review of 54 trials using DC vaccines in melanoma performed to evaluate the relationship between clinical effects and vaccine parameters, it was concluded that the objective (11.7%) and clinical (27.7%) responses for the i.n. route were the highest, but the type of response did not differ significantly among the injection routes (*p* = 0.40 and *p* = 0.64, respectively, by Kruskal–Wallis test) [125].

Another option in DC vaccination strategies to increase immunogenicity while keeping control over the anti-tumor response would be using a different subset of DCs to be loaded *ex vivo*. For instance, in some cases the use of naturally occurring DCs has been tested, such as antigen-loaded purified plasmacytoid DCs [126]. However, whether this strategy is more efficacious than the use of monocyte-derived DC vaccine remains to be determined [127]. Other groups are exploring the use of Langerhans-like cells (named “IL-15 DCs”) as sources for the DC vaccines, given their strong potential to stimulate cytotoxic T cell responses [128,129]. IL-15 DCs can be obtained by culturing monocytes from blood in IL-15 instead of IL-4 in standard protocols, and the resulting DCs have an increased capacity to stimulate NK cells cytotoxicity, which could be a crucial contribution in anti-tumor efficacy of DC vaccines [130]. In this respect, we (in collaboration with other leading groups in the field) are planning a clinical trial in ovarian cancer patients to receive an autologous vaccine comprised of selected professional crosspriming autologous dendritic cells (XP-DC), loaded *in vitro* with lysate from autologous oxidized tumor cells, administered intranodally. XP-DC are a rare subset of human myeloid DCs, representing 0.2%–0.3% of total mononuclear monocytes and expressing BDCA-3 (a.k.a. CD141 or thromobomodulin), which have been confirmed as functional equivalents to mouse CD8a+ family [131]. BDCA-3+ DC (XP-DC) are superior in cross-presentation of cell-associated antigens to CD8+ T cells to induce CTL [132,133,134], and this cross-priming capacity of XP-DC has proven essential for the initiation of effective CTL cell responses against tumors.

### 3.4. Improving the Immunocompetent Status of Vaccinated Patients

Tumor progression is generally associated with both central and peripheral tolerance mechanisms to deplete or inactivate the relevant T cell repertoire, generating an immunosuppressive tumor microenvironment (TME) allowing tumor cells to evade immune attack [66]. Some of the mechanisms elaborated by tumors that have been observed in ovarian cancer include downregulation of class I MHC molecules [135], upregulation of surface molecules that induce T cell anergy of exhaustion (*i.e*., PD-L1 [136]), releasing immunosuppressive molecules such as IDO [12], or Treg recruitment to the TME [9]. Other cells in the TME can also release factors implicated in immunosuppression, such as VEGF release by tumor vascular cells [137,138]; or myeloid-derived suppressor cells (MDSC) recruited into the TME, which may release T cell inhibitors such as arginase and nitrous oxide synthase [139]. Additionally, it has recently been identified that the critical soluble mediators of type-1 immune effector cells, IFNγ and TNFα, synergize in the induction of COX-2, the key enzyme in PGE2 synthesis, implicated in hyperactivation of MDSC within the TME of ovarian cancer patients. Interestingly, this negative feedback limiting type-1 responses could be eliminated by COX-2 blockade, allowing amplification of type-1 immunity in the TME [140].

Consistent with reduced immunocompetence associated with tumor progression, in a review of 54 trials using DC vaccination including 967 patients with melanoma, it was observed that the objective response rate did not differ significantly between stages III and IV, but the clinical response differed significantly between the two groups (*p* = 0.03), and PD cases differed significantly between stages II (18.8%) and IV (52.6%) and between stages III (23.1%) and IV (both *p* = 0.0001) [125]. Therefore, efficacy of vaccination strategies and clinical benefit could be improved by selecting patients with either minimal burden disease, or with NED (no evidence of disease) after debulking strategies, and with good performance status, whenever possible.

Additionally, most patients enrolled in clinical trials involving cancer vaccines are elderly patients with already significantly compromised immune systems, due to immunosenescence [141]. This fact could contribute to the decreased ability of the elderly to control infectious diseases, their generally poor response to vaccination, as well as to the increased incidence of cancer with age. We as well as others have demonstrated that patients with advanced ovarian cancer exhibited a dampened T-cell response to the diphtheria carrier protein CRM_197_, a potent xeno-neoantigen of Prevnar™, which was given to monitor immune responsiveness in an autologous whole tumor lysate vaccine protocol [26,142]. Interestingly, myeloma patients have previously been shown to exhibit robust T-cell responses to CRM_197_, suggesting that ovarian cancer patients may be characterized by a profound level of systemic immunosuppression. Supplementary interventions might be required to boost T cell immunity. For instance, implanting genetically engineered stromal cells in the thymus to secrete IL-7 (a T cell survival factor) has been explored in the mouse [143]. Nutritional interventions might also be useful, such as supplements of either vitamin E [144], or conjugated linoleic acid [145], as well as controlling cholesterol levels [146], which could all improve T cell function in cancer patients.

## 4. Immunomodulatory and Combinatorial Strategies in Ovarian Cancer

One way to improve vaccine efficacy would be combining them with other immunomodulatory agents aiming to obtain a synergistic effect [147,148,149]. In this respect, immune checkpoints are mechanisms established to maintain self-tolerance (therefore preventing autoimmunity), and to protect tissue from damage after immune activation in response to pathogens. Checkpoint molecules include CTLA-4 (Cytotoxic T Lymphocyte Antigen-4), PD-1 (Programmed Death-1), LAG-3 (Lymphocyte Activation Gene-3), TIM-3 (T cell Immunoglobulin and Mucin protein-3), and several others (reviewed elsewhere; [150]). They have been found to modulate T cell responses to self-proteins, but also to chronic infections and tumor antigens, with CTLA-4 being the first shown to augment antitumor immune responses [151]. Following their success in other types of immunogenic tumors [152], immunomodulatory agents are currently being tested in ovarian cancer. For instance, Hodi *et al*. reported that periodic infusions of anti-CTLA-4 antibodies after vaccination with irradiated, autologous tumor cells engineered to secrete GM-CSF (GVAX) demonstrated an objective response with durable remission for four years in one patient with ovarian cancer [153]. To clarify the role of anti-CTLA-4 as monotherapy in ovarian cancer, a new Phase II clinical trial is being conducted in the USA (NCT01611558). Other immunomodulatory agents, such as antibodies blocking PD-1/PD-L1 pathway (from different sources [154]) are currently tested; for instance, a Phase I study is being conducted to test an anti-PD-L1 moAb as monotherapy in patients with metastatic or advanced solid tumors, including ovarian cancer (NCT01772004). However, although therapies blocking the immune checkpoints show significant clinical efficacy in advanced tumors [155], attributed to potent activation of T cells, in general terms monotherapy with a single moAb yields a low rate of objective responses [156]. For this reason, the combination of vaccination with immune checkpoint blocking agents is also worth testing in clinical trials, as tumors employ both PD-1 and CTLA-4 pathways to depress the immune system. CTLA-4 and PD-1 are both coinhibitory molecules that belong to the same family of molecules, yet there is evidence suggesting that they use distinct non-redundant mechanisms to inhibit T-cell activation [157]. In preclinical models using mice with pre-implanted B16 melanomas, it has been shown that concomitant blockade of both pathways can modulate Treg functions and enhance antitumor responses, as compared to single immune checkpoint blockade [158]. In this direction, clinical trials are currently being performed to test double immune checkpoint blockade, either specifically in ovarian cancer patients (NCT02498600), or in patients with solid tumors, including patients with ovarian cancer (NCT01975831).

Interestingly, preclinical studies in ovarian cancer mouse models have demonstrated a consistently greater anti-tumor effect when checkpoint blockade and vaccines are used in combination, compared with either alone. In an ID8 ovarian cancer tumor model it was demonstrated that PD-L1 blockade can restore antitumor immunity, and synergize with whole tumor antigen vaccine (GVAX) to produce tumor rejection. Remarkably, vaccine alone was completely ineffective, while PD-L1 antibody monotherapy produced half the cure rate than GVAX plus PD-L1 blockade combination [159]. Blockade of both PD-1 and CTLA-4 in those mice resulted in reversal of CD8+ TIL dysfunction and led to tumor rejection in two-thirds of mice. Double blockade was associated with increased proliferation of antigen-specific effector CD8+ and CD4+ T cells, antigen-specific cytokine release, inhibition of suppressive functions of Tregs, and upregulation of key signaling molecules critical for T cell function [160]. Therefore, these encouraging preclinical results warrant the initiation of clinical trials trying the combination of vaccines with checkpoint inhibitors in ovarian cancer.

The IDO pathway is another regulator of the immune response in the tumor microenvironment. Indoleamine 2,3-dioxygenase (IDO) is a tryptophan-catabolizing enzyme that induces immune tolerance, by depleting tryptophan locally and producing toxic tryptophan catabolites, such as kyneurine, inhibiting proliferation of T-cells (both CD4+ and CD8+), and natural killer (NK) cells [161,162]. There is evidence showing that ovarian cancer patients with elevated IDO expression show significant impairment both in OS and PFS as compared to patients with low or no IDO expression [12]. For this reason, one IDO inhibitor, epacadostat, is currently being tested in ovarian cancer in different clinical trials, either alone as neoadjuvant treatment (NCT02042430), or in combination with checkpoint inhibitors (NCT02327078, NCT02178722) aiming to obtain a synergistic effect. One phase I/II trial (NCT02575807) is currently testing CRS-207, a recombinant Listeria-based cancer vaccine containing a live-attenuated strain of the facultative intracellular bacterium *Listeria monocytogenes* (Lm) expressing human mesothelin, in combination with epacadostat in adults with platinum-resistant ovarian cancer.

Other combinations of immunomodulatory therapies with vaccines in patients with advanced ovarian cancer include the use of Ontak (denileukin diftitox, a cytotoxic recombinant protein consisting of IL-2 protein sequences fused to diphtheria toxin) aiming to deplete CD4+CD25+ immunoregulatory T-cells (Treg) in combination with a DC based vaccine, which is currently being investigated in a Phase II study (NCT00703105). Additionally, combinatorial strategies with DC vaccination and other interventions such as radiation, chemotherapy, hyperthermia, T-cell transfer, and antibody therapy might produce important breakthroughs in treating patients with ovarian cancer. There is growing evidence that radiation therapy targeted to the tumor can convert it into an *in situ* tumor vaccine by inducing release of antigens during cancer cell death in association with pro-inflammatory signals that trigger the innate immune system to activate tumor-specific T cells [163]. Radiation also affects the tumor microenvironment allowing increased infiltration by activated T cells and overcoming some of the mechanisms of tumor immunosuppression. Recently, in a pilot clinical trial including patients with metastatic solid tumors, the combination of radiotherapy with GM-CSF produced objective abscopal responses (radiotherapy-induced immune-mediated tumor regression at sites distant to the irradiated field) in some patients (26.8%) [164]. Additionally, it has been observed that including TGFβ blockade and anti-PD-1 antibodies extended survival achieved with radiation [165]. Further strategies using combinations of radiotherapy and immunotherapy are therefore currently being explored [166,167]. In ovarian cancer, although radiation is not widely accepted as a routine treatment modality in the initial treatment in EOC patients, it can be considered in higher-risk stage I and II disease and in stage III disease where small-volume residual disease is present after surgery.

The potential of chemotherapy combination with DC vaccination has also been explored. In a pilot clinical study including seven stage III colon cancer patients receiving standard adjuvant oxaliplatin/capecitabine chemotherapy and vaccinated at the same time with keyhole limpet hemocyanin (KLH) and carcinoembryonic antigen (CEA)-peptide pulsed DCs, an enhanced non-specific T-cell reactivity upon oxaliplatin administration was observed, results that support further testing of the combined use of tumor vaccination with oxaliplatin-based chemotherapy [168]. Importantly, platinum-based chemotherapy is the mainstay of treatment in EOC, and therefore chemotherapy should be considered in combination strategies to improve vaccine efficacy in ovarian cancer treatment.

Other immunomodulatory agents could also enhance antitumor responses in DC-based immunotherapy. It was recently described that the infiltration of T cells into the tumor endothelial barrier was mediated by the death mediator Fas ligand (FasL/CD95L) in the tumor vasculature of human and mouse solid tumors [169] and it was demonstrated that tumor-derived VEGF-A, IL-10 and prostaglandin E (PGE) cooperatively induced FasL expression in endothelial cells, allowing them to kill effector CD8+ T cells but not Treg cells, which express higher levels of c-FLIP. Dual inhibition of VEGF and PGE with anti-VEGF and aspirin, demonstrated a significant effect in tumor regression. These observations led us to conclude that modulating the tumor endothelial barrier with aspirin and bevacizumab is a promising approach to combine with vaccinations. Thereby, we conducted a clinical trial for subjects with recurrent ovarian cancer using OCDC (DC loaded with oxidized tumor lysate) administered intranodally alone, or in combination with i.v. bevacizumab and cyclophosphamide (aiming to reducing Tregs), or in combination with i.v. bevacizumab, cyclophosphamide and aspirin (NCT01132014) and the results are currently being analyzed.

## 5. Concluding Remarks

**Figure 1:**
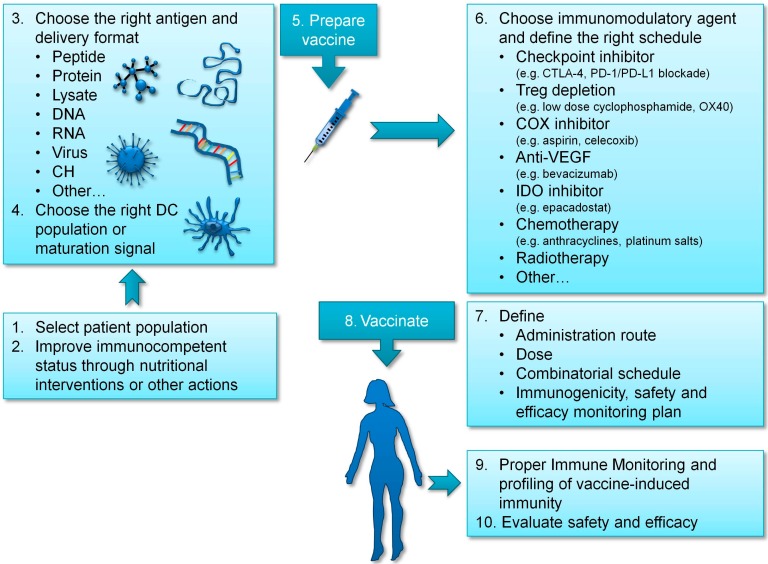
Summary of required considerations to implement a successful vaccination strategy in ovarian cancer.

At present, a plethora of different options is open to discovery in the treatment of ovarian cancer. This represents a great opportunity to improve patients’ condition, and we can be reasonably optimistic that some of those options will surely translate into clinical benefit for patients. However, new opportunities come always together with new challenges, which we will have to confront and overcome. Among these, the first obvious challenge is to choose the options with more chances to yield clinical benefit, which requires a sound understanding of deep biological processes engaged in immune response to tumor development, and this implicates continuous investment in fundamental research. DC vaccines are one of the most promising options, due to a currently acceptable objective response rate, comparable to that obtained using standard of care approaches or checkpoint inhibitors, which has been demonstrated in different indications [85], coupled to a good safety profile (rarely presenting immune-related toxicities [60]), and associated with preserved quality of life of cancer patients [61]. Nevertheless, as we have previously discussed, results could be further improved by carefully selecting the right antigen to target, the right DC subtype, and the adequate adjuvant and delivery route. In addition, choosing the right patient population to vaccinate and improving patients’ immunocompetent status could also contribute to increased clinical benefit (see Figure 1).

Finally, using combinatorial approaches aiming to obtain synergistic effects could lead to major breakthroughs in treating patients with ovarian cancer. However, it should be taken into account that some options will be limited by technical capacities, being restricted to centers with the right infrastructure and finances to successfully develop these strategies. This will undoubtedly generate deep discussions regarding patients’ access to treatment. From the clinical point of view, although combination of DC vaccines with other agents can provide long-term benefit to incurable patients, this has to be tempered by the observation that patients may present severe (even lethal in some cases) auto-inflammatory events, mostly in the lungs and in the gastrointestinal tract, that need to be early recognized by oncologists to allow effective management, implicating close monitoring of patients during and after treatment, as it is the case for checkpoint inhibitors [170]. Furthermore, it has to be noted that clinical trials design in immuno-oncology needs to be carefully planned, as safety and efficacy concerns differ substantially from those evaluated in clinical trials with cytotoxic agents [171]: immuno-oncology agents present unique kinetics, compared with traditional targeted or chemotherapeutic agents [172], and their combination require a rational design of synergistic combination strategies [173]. Finally, a detailed strategy for early detection and management of toxicity as well as planned demonstration of predicted additive or synergistic benefit are also required.

## Figures and Tables

**Table 1:** Published results from therapeutic vaccines tested in ovarian cancer from 2000 to date.

Vaccine Class	Vaccine Name	Description	Clinical Development: Phase	No. of Pts (OvCa Pts)	Clinical Outcome
DCs	APCEDEN	DCs loaded with whole-tumor lysate	Phase II; (Bapsy, 2014 [24])	38 pts (9 OvCa pts)	No CR observed; ORR was 28.9% (11/38) and irRC was 42.1% (16/38)
	OCDC	DCs loaded with autologous oxidized tumor lysate	Pilot; (Chiang, 2013 [25])	5 OvCa pts	2/5 pts (40%) demonstrated PFS2 > PFS1
	DCVax-L	DCs loaded with autologous oxidized tumor lysate, combined with bevacizumab and metronomic Cy	Pilot; (Kandalaft, 2013 [26])	6 OvCa pts	4/6 pts (66%) achieved clinical benefit (including 2 PR and 2 SD)
	DC-wtl	DCs loaded with crude whole tumor lysate	Phase I; (Hernando, 2002 [27])	8 pts (6 OvCa pts)	Data suggested a positive correlation with disease stabilization
	DC-MFP	DCs loaded with mannan-MUC1 fusion protein (MFP)	Phase I; (Loveland, 2006 [28])	9 pts (2 OvCa pts)	2/9 pts (22%) in progression at entry were stable after therapy, for at least 3 years
	Lapuleucel-T, Neuvenge, APC 8024	DCs loaded with BA7072, a fusion protein HER-2/neu linked to GM-CSF	Phase I; HER-2+ tumors; (Peethambaram, 2009 [29])	18 pts (4 OvCa pts)	2/18 pts (11%) had SD lasting > 48 weeks
	HER-2/neu; MUC1 peptides	DCs loaded with synthetic peptides derived from HER-2/neu or MUC1 peptides	Phase I; HER-2+ or MUC1+ tumors; (Brossart, 2000 [30])	10 pts (3 OvCa pts), HLA-A*02+	No data
	hTERT; HER-2/neu; PADRE peptides	DCs loaded with synthetic peptides derived from hTERT; HER-2/neu; PADRE	Phase I/II; (Chu, 2012 [31])	14 OvCa pts, HLA-A*02+	3 years-OS was 90%; 3 years-PFS was 80% (with Cy)
	WT-1; MUC1; CA125	DCs loaded with synthetic peptides derived from WT-1; MUC1; CA125	Phase II; (Kobayashi, 2014 [32])	56 OvCa pts	DCR and ORR were 29% and 3.6%, respectively
Whole tumor cells	Fang vaccine, Vigil™ Ovarian, Gemogenovatucel-T	Autologous tumor cells eletroporated with FANG vector, a plasmid encoding GM-CSF and a bi-shRNA targeting furin convertase, thereby downregulating TGF-b1 and b2	Phase I; (Senzer, 2012 [33])	27 pts (5 OvCa pts)	23/26 pts (88%) showed SD at month 2 or later
*Listeria monocy togenes*	CRS-207	Lm strain engineered to express human mesothelin	Phase I; (Le, 2012 [34])	17 pts (2 OvCa pts)	37% of subjects lived ≥ 15 mo (months)
Peptides/proteins	Mixture of peptides (comparison)	Predesigned peptides *vs.* PPV (personalized peptide vaccine); admixed with Montanide ISA-51	Pilot; (Tsuda, 2004 [35])	14 pts (5 OvCa pts), HLA-A*02+ or HLA-A*24+	No clinical response with predesigned; 3/5 cervical cancer pts (60%) showed objective tumor regression
	Mixture OvCa-associated peptides	OvCa-associated peptides admixed with Montanide ISA-51 and GM-CSF	Pilot; (Morse, 2011 [36])	15 pts (8 OvCa pts); HLA-A*02+	With median follow-up of 492 days, 4 OvCa pts had relapsed and 3 died (expected relapse rate 18–22 mo in 75% of pts)
	Mixture of different peptides	OvCa-associated peptides plus a helper peptide from tetanus toxoid protein, admixed with Montanide ISA-51 and GM-CSF	Phase I; (Chianese-Bullock, 2008 [37])	9 OvCa pts, HLA-A*01+, -A*02+ or A*03+	One participant remained disease-free at 19 months after active treatment
	HER-2/neu	Epitope p369–377, admixed with GM-CSF	Phase I; HER-2/neu++ Tu (Knutson, 2002 [38])	6 pts (2 OvCa pts), HLA-A*02+	No data
	-	Multiple peptides derived from either the extracellular domain (ECD) or the ICD, admixed with GM-CSF	Phase I; HER-2/neu++ Tu (Disis1, 2002 [39])	38 pts (5 OvCa pts), HLA-A*02+	No data
	-	Peptides from the ICD, admixed with GM-CSF	Phase I; HER-2/neu++ Tu (Disis, 2002 [40])	10 pts (1 OvCa pts)	No data
	-	Multiple peptides derived from either the ECD, the ICD, or both, admixed with GM-CSF	Phase I; HER-2/neu++ Tu (Disis, 2004 [41])	38 pts (5 OvCa pts)	No data
	HER-2/neu-ICD	ICD protein, aas 676–1255, His-tagged	Phase I; HER-2/neu++ Tu (Disis, 2004 [42])	29 pts (1 OvCa pt)	No data
	NY-ESO-1	Epitope p157–170, admixed with Montanide ISA-51	Phase I; (Odunsi, 2007 [43])	18 OvCa pts, HLA-DPB1*0401+ or *0402+	Median PFS of 19.0 mo (*vs.* 16–18 weeks in pts receiving 2nd line chemo)
	-	Epitope p157–165, admixed with Montanide ISA-51	Phase I; NY-ESO-1+ or LAGE-1+ Tu; (Diefenbach, 2008 [44])	9 OvCa pts, HLA-A*02:01+	Median PFS of 13 mo. 3/9 pts (33%) remained in CR at 25, 38, and 52 mo
	NY-ESO-1 OLP	NY-ESO-1 overlapping long peptides, +/− Montanide and Poly-ICLC	Phase I; (Sabbatini, 2012 [45])	28 OvCa pts (HLA indep)	Pts NY-ESO-1+ receiving OLP + Montanide + Poly-ICLC showed delayed time to recurrence
	NY-ESO-1 protein	NY-ESO-1 protein + Montanide + CM-CSF +/− decitabine	Phase I; (Odunsi, 2014 [46])	12 OvCa pts	5/10 (50%) pts had SD (median duration 6.3 mo), and 1/10 (10%) had PR (duration 5.8 mo)
	P53	Wt p53: 264–272 peptide admixed with GM-CSF and Montanide ISA-51, either SC (Arm A) or loaded into DCs (Arm B)	Phase II; p53++ Tu; (Rahma, 2012 [47])	21 OvCa pts, HLA-A*02:01+	No significant difference between arms in median OS (40.8 mo *vs.* 29.6 mo, *p* = 0.26), nor in PFS (4.2 mo *vs.* 8.7 mo, *p* = 0.94)
	P53-SLP	Ten synthetic peptides 25–30 aa long overlapping peptides (aas 70–248 in wt-p53) admixed in Montanide ISA-51	Phase II; (Leffers, 2009 [48])	18 OvCa pts (HLA indep)	2/18 (11%) of pts with SD, not clearly attributable to vaccination
	-	-	Phase II; (Leffers, 2012 [49])	20 OvCa pts (HLA indep)	No difference in survival between p53-SLP treated pts and historical controls (median 44.0 mo *vs.* 47.4 mo, *p* = 0.601)
	-	Same, but two days before vaccination, 300 mg/m^2^ Cy i.v. was given	Phase II; (Vermeij, 2012 [50])	10 OvCa pts (HLA indep)	No data
	PPV	Personalized peptide vaccine: mixture of 4 peptides (from a panel of 31) previously tested for immunity in each pt, admixed in Montanide ISA51VG	Phase II; (Kawano, 2014 [51])	42 OvCa pts (HLA-dep)	Median survival time (MST) was 39.2 mo in platinum-sensitive pts, *vs.* 16.2 mo in platinum-resistant
	Flt3-L	Truncated glycoprotein Flt3-L (Fms-like tyr kinase-3-ligand, which increases DCs and monocytes), either i.p. or s.c.	Pilot; (Freedman, 2003 [52])	15 pts (9 OvCa pts)	No objective responses were observed
Genetic vaccines	PANVAC-C + PANVAC-V	Poxviral vaccine: CEA-MUC1-TRICOM (B7.1, ICAM-1, LFA-3) engineered into vaccinia (PANVAC-V) as prime and fowlpox (PANVAC-C) as booster vaccination	Pilot; CEA+ or MUC1+ Tu; (Gulley, 2008 [53])	25 pts (3 OvCa pts)	1 OvCa pt (1/25: 4%) had durable (18 mo) clinical response
	rV-NY-ESO-1 + rF-NY-ESO-1	NY-ESO-1 engineered into vaccinia (rV) as prime and fowlpox (rF) as booster vaccination	Phase I; NY-ESO-1+ Tu; (Jager, 2006 [54])	36 pts (1 OvCa pt)	7/9 pts with stage II/IV MEL survived 17–63+ mo
	-	-	Phase II; NY-ESO-1+ Tu; (Odunsi, 2012 [55])	47 pts (22 OvCa pts)	In OvCa pts, median TTP was 21 mo and median OS was 48 mo
Epigenetic vaccines	Theratope^®^	Synthetic Syalyl-Tn-KLH (STn: carbohydrate associated with the MUC1 mucin), admixed with Detox-B, after autologous transplantation	Phase II/III; MUC1+ Tu; (Holmberg, 2003 [56])	70 pts (17 OvCa pts)	Decreased risk for relapse and death (*p* = 0.07 and *p* = 0.1 respectively), as compared to transplanted pts only
	Lewis(y)	Synthetic Lewis(y) pentasaccharide coupled to KLH (Ley: carbohydrate epitopes overexpressed in OvCa), admixed with QS-21	Phase I; (Sabbatini, 2000 [57])	25 OvCa pts	Median TTP was 6 mo (2–17 mo)

Abbreviations: aas, aminoacids; CR, complete response; DCR, disease control rate (SD + PR + CR); irRC, immune-related response criteria; mo, months; MST, median survival time; ORR, objective response rate (PR + CR); OS, overall survival; PD, progressive disease; PFS, progression free survival; PR, partial response; Pt(s), patient(s); SD, stable disease; TTP, time to progression. Source: PubMed search using the terms “ovarian cancer clinical trial” plus “vaccine” or “active immunotherapy”, manually selecting the relevant publications. Note: the HLA serotypes have been adapted to the new nomenclature established on 2010 by the WHO Naming Committee for Factors of the HLA System [58].

**Table 2:** Vaccines for ovarian cancer in clinical development (January 2016).

Type	Product Name	Description	Clinical Development: Phase Indication NCT
DC	DCVAC/OvCa	DCs activated with an ovarian tumor cell lysate	Phase II OC NCT02107378
	FRalphaDC vaccine	DCs loaded with five immunogenic peptide epitopes, derived from the tumor-associated antigen human folate receptor alpha (FR alpha or FOLR1), including FR30, FR56, FR76, FR113, and FR238	Pilot OC NCT02111941
	Ontak + DC vaccine	Ontak (denileukin diftitox): a cytotoxic recombinant protein consisting of interleukin-2 (IL-2) protein sequences fused to diphtheria toxin; the use of Ontak is followed by autologous DC vaccine to stimulate tumor killing immune cells	Phase II OC NCT00703105
	Ovapuldencel-T	DCs loaded with autologous, lethally irradiated cancer cells and mixed with GM-CSF	Phase II OC NCT02033616
	Dendritic cell/tumor fusion vaccine	DC/tumor fusion vaccine with GM-CSF and imiquimod (cytokine production stimulation)	Phase II OC NCT00799110
Whole tumor cells	Fang vaccine, Vigil™ ovarian, Gemogenovatucel-T	Autologous tumor cells eletroporated with FANG vector, a plasmid encoding GM-CSF and a bi-shRNA targeting furin convertase, thereby downregulating transforming growth factor (TGF)-β1 and β2	Phase II/III OC NCT02346747
Peptide/protein	OVax	Autologous ovarian cancer cell peptide antigens conjugated to the hapten 2,4-dinitrophenol (DNP)	Phase I/II OC NCT00660101
	HER-2 peptide vaccine	Combination of MVF-HER-2 (597–626) and MVF-HER-2 (266–296) emulsified with nor-MDP and ISA 720	Phase I Solid TumorsNCT01376505
	FBP E39/J65	Two Folate Binding Protein Peptide Vaccines (E39 and J65)	Phase I/II BC/OC NCT02019524
	WT2725	Peptide derived from Wilms tumor gene 1 (WT1) protein	Phase I WT1++ Tumors NCT01621542
	DSP-7888 Dosing emulsion	WT1 protein-derived peptide vaccine	Phase I Different Tumors NCT02498665
	OC-L	Trial to test the addition of 2 investigational agents, Montanide and poly-ICLC (a TLR3 agonist) to a backbone of autologous oxidized tumor cell lysate vaccine (OC-L) administered with GMCSF	Phase I OC NCT02452775
Genetic	Ad-sig-hMUC-1/ecdCD40L	Ad-sig-hMUC-1/ecdCD40L adenoviral vector encodes a fusion protein in which the hMUC-1 epithelial antigen is attached to the CD40L (CD40 ligand), which binds to CD40 on DCs, stimulating internalization of hMUC-1 Ag	Phase I Epithelial Ca (LC/BC/OC/PC/CRC) NCT02140996
	AdV-tk + valacyclovir	AdV-tk: adenoviral vector expressing the herpes simplex virus thymidine kinase (HSV-tk) gene, which, when administered in conjunction with a synthetic acyclic guanosine analogue (valacyclovir), possesses potential antineoplastic activity. Release of TAAs by dying tumor cells may then stimulate an antitumor cytotoxic T lymphocyte (CTL) response	Phase I Epithelial Ca (LC/MES/BC/OC) NCT01997190
	ID-LV305	ID-LV305: An engineered lentiviral vector targeting DCs and containing nucleic acids encoding for the human tumor-associated cancer-testis antigen NY-ESO-1	Phase I MEL/NSCLC/OC/SAR NCT02122861
	p53MVA	p53MVA vaccine: modified vaccinia virus Ankara expressing tumor protein p53	Phase I OC NCT02275039
	Trovax^®^	Trovax^®^: modified vaccinia virus Ankara (MVA) vector, encoding the 5T4 antigen	Phase II OC NCT01556841
Epigenetic	OPT-822/OPT-821	OPT-822/OPT-821: Two carbohydrate-based immunostimulants comprised of the Globo H hexasaccharide 1 (Globo H) epitope linked to KLH, which may stimulate a cytotoxic T-lymphocyte (CTL) response against Globo H-expressing tumor cells	Phase II OC NCT02132988

Abbreviations: BC, breast cancer; CRC, colorectal cancer; CTLA-4, cytotoxic T-lymphocyte-associated antigen 4; i.p., intraperitoneal; IDO1, indoleamine 2,3-dioxygenase; LC, lung cancer; MEL, melanoma; MES, mesothelioma; moAb, monoclonal antibody; mOC, metastatic ovarian cancer; NSCLC, non-small cell lung cancer; OC, ovarian cancer; PCRC, pancreatic cancer; PC, prostate cancer; PD-(L)1, programmed death-(ligand)1; ROC, recurrent ovarian cancer; SAR, sarcoma; TAA, tumor-associated antigen; VEGF(R), vascular endothelial growth factor (receptor); Note: This is not a complete list of all immunotherapies in clinical development in ovarian cancer. Source: ClinicalTrials.gov.

## Abbreviations

The following abbreviations are used in this manuscript:

BC: breast cancer
CEA: carcinoembryonic antigen
CRC: colorectal cancer
CTLA-4: cytotoxic T-lymphocyte-associated antigen 4
DC: dendritic cells
EOC: epithelial ovarian cancer
GM-CSF: granulocyte-macrophage colony-stimulating factor
i.p.: intraperitoneal
IDO1: indoleamine 2,3-dioxygenase
IFN: interferon
KLH: keyhole limpet hemocyanin
LC: lung cancer
MDSC: myeloid derived suppressor cells
MEL: melanoma
MES: mesothelioma
MHC: major histocompatibility complex
moAb: monoclonal antibody
mOC: metastatic ovarian cancer
NED: no evidence of disease
NSCLC: non-small cell lung cancer
OC: ovarian cancer
OCDC: DC loaded with oxidized tumor lysate
PCRC: pancreatic cancer
PC: prostate cancer
PD-(L)1: programmed death-(ligand)1
PGE: prostaglandine E
PPV: personalized peptide vaccine
ROC: recurrent ovarian cancer
SAR: sarcoma
shRNA: short hairpin RNA
TAA: tumor-associated antigen
TGF: transforming growth factor
TIL: tumor infiltrating lymphocytes
TME: tumor microenvironment
Tregs: regulatory T cells
VEGF(R): vascular endothelial growth factor (receptor)
